# Radical Prostatectomy: Hospital volumes and surgical volumes – does practice make perfect?

**DOI:** 10.1186/1471-2482-9-10

**Published:** 2009-06-06

**Authors:** Cydney Urbanek, Ryan Turpen, Charles J Rosser

**Affiliations:** 1 Department of Urology, University of Florida, Gainesville, Florida, USA

## Abstract

**Background:**

Between the years 1993 and 2003, more than 140,000 men underwent radical prostatectomy (RP), thus making RP one of the most common treatment options for localized prostate cancer in the United States.

**Discussion:**

Localized prostate cancer treated by RP is one of the more challenging procedures performed by urologic surgeons. Studies suggest a definite learning curve in performing this procedure with optimal results noted after performing >500 RPs. But is surgical volume everything? How do hospital volumes of RP weigh in? Could fellowship training in RP reduce the critical volume needed to reach an 'experienced' level?

**Summary:**

As we continue to glean data as to how to optimize outcomes after RP, we must not only consider surgeon and hospital volumes of RP, but also consider training of the individual surgeon.

## Background

One out of every six men in the US will be diagnosed with prostate cancer making it the second most commonly diagnosed cancer among American males over the age of 45 years [1]. The most common treatment for localized prostate cancer remains radical prostatectomy (RP) [2]. Over the past 30 years, RP has significantly transformed from what Dr. Hugh Hampton Young first described in 1904. Early prostatectomies were fraught with significant morbidity and mortality, until Walsh and others reported on the anatomic, nerve sparing RP in 1982. The anatomic, nerve sparing RP was associated with less blood loss and reduced incidence of erectile dysfunction [3]. Several reports documented more favorable outcomes in physicians who had performed more than 500 RPs. For example, in Catalona et al's report of 1,870 men undergoing RP, postoperative complications were less likely with increasing surgeon experience [4].

Prostatectomy is a challenging radical surgery performed in appropriately selected men for prostate cancer. Though cancer control is the key for this procedure, we must strive for favorable post operative quality of life outcomes (e.g., urinary continence, bowel continence, and erectile function). More so than with other genitourinary malignancies, these quality of life outcomes are extremely important since some of these tumors are non-life threatening.

Previous research has linked outcomes from RP to hospital volume, surgeon volume and the level of surgical training. Many studies suggest that these volume-outcome relationships exist because of the perceived favorable trends associated with volumes and outcomes (i.e., outcomes must inevitably improve as surgeon's and hospital's volume increase) [5]. Prompted by such reports, this manuscript aims to review literature related to training and volume as it relates to outcomes of RP.

## Discussion

### Hospital volume

Numerous studies have evaluated the relationship between the number of RPs performed at hospitals to the clinical outcomes [3,4]. These studies have varied significantly in the hospital volumes. Currently, there is no universally accepted definition for what is considered to be a high or low volume center. Table 1 shows the distribution of some of these studies that assessed RP outcomes based on hospital volume. Results from these retrospective studies are mixed in regards to outcomes and have used various endpoints to critically evaluate differences in these centers. Outcomes measured have included everything from length of stay to mortality. Whereas mortality associated with RP should not be the sole endpoint to monitor since the overall reported mortality rate for the procedure is < 1%, other endpoints such as oncologic outcomes and quality of life related outcomes have significant implications in patient care [6].

**Table 1:** Studies of effect of hospital and surgeon volume on outcomes

**Hospital Volumes**							
**Hu et al.**		< 60 RP/yr			> 60 RP/yr		
**In hospital complications adjusted OR (95% CI)**		1.0			0.84 (0.59–1.19)		0.3
**Length of stay parameter estimate (95% CI)**		1.0			-0.42 (-0.89–0.03)		0.08
**Anastomatic stricture rate adjusted OR (95% CI)**		1.0			0.72 (0.49–1.04)		0.09
**Begg et al**	Low (1–33)		Medium (34–61)	High (62–107)		Very High (114–252)	
**% 30 day mortality**	0.5		0.5	0.5		0.5	0.8
**% 60 day mortality**	0.6		0.6	0.6		0.5	0.7
**% post-op complications**	32		31	30		27	0.03
**% late urinary complication**	28		29	23		20	0.01
**% long-term incontinence**	19		19	18		18	0.2
**Ellison et al**	Low, < 25 RP/yr		Medium, 26–54 RP/yr	High, > 54 RP/yr			
**In hospital mortality adjusted OR (95% CI)**	1.78 (1.2–2.7)		1.71 (1.2–2.6)	1.0			<0.001
**Ave length of stay (days)**	7.3		-	6.1			<0.0001
**Yao et al**	Low, < 38/yr		Medium-low, 39–74 RP/yr	Medium high, 75–140		High > 140 RP/yr	
**% 30 day mortality (95% CI)**	0.63 (0.53–0.73)		0.59 (0.49–0.68)	0.56 (0.47–0.66)		0.39 (0.31–0.46)	0.0015
**% 60 day mortality (95% CI)**	5.0 (4.7–5.3)		4.5 (4.3–4.8)	4.3(4.0–4.5)		4.1 (3.8–4.3)	0.03
**% overall complications (95% CI)**	31.3 (30.8–31.9)		28.7 (28.2–29.3)	27.8 (27.2–28.3)		26.3 (25.8–26.9)	0.02
**Mean day length of stay (95% CI)**	8.51 (8.47–8.56)		8.18 (8.14–8.22)	7.70 (7.66–7.74)		7.81 (7.77–7.85)	0.0001
**Surgeon Volume**							
**Hu et al.**		Low, < 40 RP/yr		High, > 40 RP/yr			
**In hospital complications adjusted OR (95% CI)**		1.0		0.53 (0.32–0.89)			0.02
**Length of stay parameter estimate (95% CI)**		1.0		0.68 (1.26–0.06)			0.03
**Anastomatic stricture rate adjusted OR (95% CI)**		1.0		0.89 (0.55–1.44)			0.6
**Begg et al**	Low 1–10 RP/yr		Medium 11–19 RP/Yr	High 20–32 RP/yr		Very High, >33 RP/yr	
**% 30 day mortality**	0.4		0.5	0.5		0.5	0.7
**% 60 day mortality**	0.5		0.5	0.6		0.6	0.6
**% post-op complications**	32		31	30		26	<0.001
**% late urinary complication**	28		26	27		20	0.001
**% long-term incontinence**	20		20	19		16	0.04

(Modified, Nuttall, J Urol 2004)

In one study, high volume hospitals were associated with reduction in length of stay and in-hospital mortality [7]. However, Begg and others noted no significant difference in mortality between low and high volume hospitals. This study did demonstrate a higher risk of post-operative urinary complications at low volume hospitals [8]. Similarly, Hu and colleagues showed that high volume hospitals reported fewer urethral anastomatic strictures while low volume centers had an increased stricture rate. In regards to long-term incontinence, despite the significant difference in stricture rates, there was no statistically significant difference between low volume and high volume centers [9]. Furthermore, in a large study from England comprised of over 18,000 men who underwent RPs, there was only a slight difference in the rate of post-operative complications between low volume and high volume centers, 2.3% vs. 1.2%, respectively [10].

Confounding many of these studies comparing high and low volume centers is the fact that high volume centers tend to see more high surgical risk patients and high risk prostate cancer patients in regards to grade and stage of the disease being treated compared to the low volume centers [11]. Many hospitals have implemented clinical care pathways as a means to reduce hospital stay, post-operative complication, and overall cost. After implementing the clinical care pathway, Chang and others shorten length of stay from 3 days to 2 days without compromising the quality of care [12]. In addition, regionalization of RP from low volume to high volume hospitals can also decrease the length of stay post RP [1]. Ellison and colleagues sought to identify a relationship between hospital RP volume and oncologic outcomes. They reported that in low volume hospitals, 25% more of the patients deemed to have low risk prostate cancer (i.e., low grade and low stage disease) require some form of post operative adjuvant therapy [11]. It must be noted that these studies that report on volumes are heterogeneous with varied methodology in data analysis.

Volume-outcome studies done by general surgery or gynecology such as pyloromyotomy, pancreatectomy, esophagectomy, pneumonectomy, liver resection, and pelvic exenteration have shown compelling results that support the theory that high volume hospitals offer better quality of care than low volume hospitals [13,14]. One must remember that the high volume hospitals may have access to state of the art equipment and facilities as well as more staff with specialty training. With this in mind, healthcare providers may be more likely to refer their patients to these better equipped, high volume hospitals expecting superior outcomes.

### Surgeon volume

Several reports documented more favorable outcomes in surgeons who have performed more than 500 RPs. For example, Catalona reported his results of 1,870 men undergoing RP and found postoperative complications were less likely with increasing surgeon experience [4]. Recently, two large studies reported patient outcomes as related to surgeon's volumes [15,16]. Neither study reported an increase in the relative risk of surgery related death associated with surgeon volume. Taking into consideration patient age and comorbidities, an increase risk of post-operative complications was noted among surgeons who performed fewer than 40 RP per year. Even more startling was a significantly higher transfusion rate when surgeons performed less than 15 RP per year. Furthermore, surgeons who performed more than 15 RP per year had shorter hospital length of stay [15,16].

Regarding quality of life outcomes and oncologic outcomes, association with surgeon volumes is varied. In a study by Begg and others, high volume surgeons, defined as those who performed a minimum of 20 procedures during the five-year study, had fewer late urinary complications. However, there was no difference in the rate of positive surgical margins between the surgeons [8]. Similarly, Chun and others reported the association of surgical margins in 2,402 men with localized prostate cancer treated with RP by 11 highly trained surgeons. High volume surgeons were noted to have a statistically significant lower rate of positive surgical margin (18.9% vs. 22.6%). However, when assessed in a multivariate analysis with low and intermediate volume surgeons, the association between a reduction of positive surgical margins in high volume surgeons diminished [15], thus possibly illustrating the importance of another factor (e.g., surgical training) in this complex procedure.

**Figure 1:**
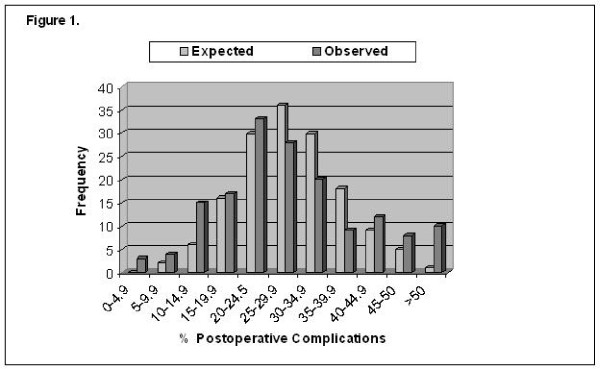
**Histograms juxtapose observed and expected number of surgeons' postoperative complications**. More outliers on right side of histogram in observed vs. expected plot highlight providers achieving poor outcomes. On the other hand, outliers toward left in observed vs. expected plots indicate surgeons achieving more favorable outcomes (From Bianco, J Urol, 2005, copyright permission).

**Figure 2:**
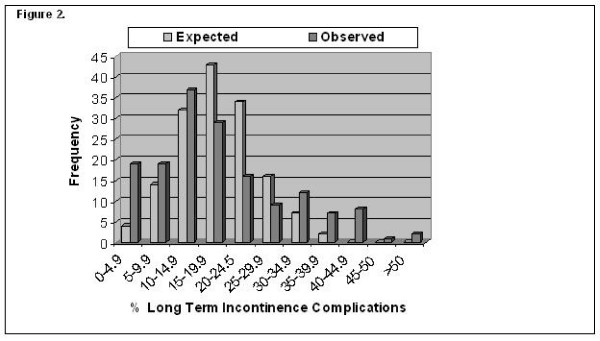
**Histograms juxtapose observed and expected number of surgeons' long-term incontinence**. More outliers on right side of histogram in observed vs. expected plot highlight providers achieving poor outcomes. On the contrary, outliers toward left in observed vs. expected plots indicate surgeons achieving more favorable outcomes (From Bianco, J Urol, 2005, copyright permission).

In a study by Bianco and colleagues, 10,737 patients who underwent RP by 999 surgeons were analyzed. In this study, expected outcomes in major post-operative complications, late urinary complications and long-term incontinence were compared with the observed outcomes of these three domains. For all three outcomes, the variation among surgeons in the rate of complications was significantly greater than that expected by chance after adjustment of covariates. Furthermore, surgeons with better results with regard to one outcome were likely to have better results with regard to the other two outcome measures (Figures 1 &2). The authors concluded that outcomes might very well be related to surgical technique and not strictly surgical volume [16]. In a sense, they are implying that practice does not necessarily make perfect.

But if a surgeon's volume is truly felt to be the major culprit in suboptimal outcomes, perhaps a minimum volume threshold (MVT) should be required for difficult cases. Three hundred and seven urologists were queried as to their thoughts on a MVT for such procedures as radical nephrectomy, radical cystectomy and radical prostatectomy. The majority of the surgeons were in favor of MVTs. Interestingly enough, the most complex surgeries were given the lowest MVT based on their frequency. For example, radical cystectomy with continent urinary diversion had the lowest MVT (1–5/yr), while RP, considered to be the third most complex surgery, was given the highest MVT (>20/yr) [17]. With this said, volume thresholds based on frequency of procedure instead of complexity of procedure may not correlate with the best outcomes for patients due to the fact that increasing volume does not guarantee improvements in outcome since low and high volume surgeons can produce both good and poor outcomes.

### Fellowship Training

Everything that has been discussed so far hinges on the premise that practice makes perfect (or increase volume leads to superior outcomes). Previous reports from experienced surgeons reporting on pathologic outcomes, and overall complication rates hinted that as many as 200–500 RPs must be performed before a surgeon reaches the expert portion of the learning curve [4,18,19]. Though attention should be on volumes, perhaps there are other factors that can influence outcomes, specifically additional training (i.e., urologic oncology fellowship).

The ultimate goal of RP is cancer control with little to no morbidity. The specialized training obtained during a urologic oncology fellowship affords surgeons an opportunity to study in depth the art and science of prostate cancer treatment and thus achieve cancer control rates and surgical outcomes similar to those of more experienced surgeons. In a recent study, two recently graduated urologic oncologists reported the outcomes of their first 66 RP. Operative time, estimated blood loss, transfusion rates, and post-operative complications [20] were similar to those previously reported by more experienced surgeons [21,22]. Furthermore, postoperative complications were similar in nature and frequency to those previously documented in the literature. More important, the pathologic outcomes (i.e., the ability to correctly perform this oncologic procedure) were similar to those previously reported by more experienced surgeons. On the basis of the study reported here, it is believed that the patient volume and practice necessary to achieve the outcomes seen by more experienced surgeons may be obtained during a urologic oncology fellowship training program.

Recently, Klein and others reported the results of a collaborative study which hypothesized that surgeon's experience is more important to ensuring favorable outcomes than certain preoperative patient risk factors (serum PSA, clinical stage, and Gleason score). Their results showed a statistically significant association between biochemical recurrence and surgeon's experience such that patients receiving treatment from a surgeon with 10 versus 250 prior RPs would have an absolute risk difference of 6.6%, 12.0% and 9.7% for low, intermediate, and high risk prostate cancer. Moreover, the data illustrated that the most experienced surgeons had an almost zero percent 5-year biochemical relapse rate for low risk prostate cancer patients. Such a substantial cancer control rate suggests that the primary cause for recurrence would most likely be poor surgical technique [23].

The notion of better outcomes observed in fellowship trained practitioners is not unique to the treatment of prostate cancer. In fact, the gynecological and general surgical literature have extensively reported on this concept [24-28]. Thus, being fellowship trained in an environment where an experienced surgeon serves as first assistant to the trainee in a manner committed to perfect his/her skills, might be one way to reduce the surgeon volume or MVT needed to obtain acceptable outcomes with RP.

## Summary

A look in the literature regarding the learning curve for prostatectomies clearly illustrates that surgical volume, hospital volume, and surgical training are integral to successful outcomes. Some have even defined a MVT of 200–500 cases that must be performed before one achieves the expertise level. The importance of dedicated oncologic fellowship training would seemingly become an integral, if not necessary ingredient in developing experienced prostatectomists, capable of performing RPs with maximal outcomes.

Although there is increasing evidence that high volume hospitals and high volume surgeons produce more favorable outcomes, caution is still advised when interpreting the overall results. Most short-term outcomes such as hospital length of stay and post operative complications are reduced in settings of a high volume center/surgeon; however, the real concern lies within the long-term outcomes, i.e., cancer control, continence and potency. Even in experienced hands, achieving successful outcomes in all three of these domains is extremely difficult. However, initiatives are in the pipeline to link procedural reimbursements with the performance (or outcomes) associated with the surgeons. This is more impetus for surgeons to critically review their outcomes to improve their performance and maximize patient benefit.

## Abbreviations

RP: radical prostatectomy; MVT: minimum volume threshold.

## Competing interests

The authors declare that they have no competing interests.

## Authors' contributions

CU collected data and drafted the manuscript. RT assisted in collecting data and revising the manuscript. CJR conceived the project, and participated in its design and coordination. All authors read and approved the final manuscript.

## Pre-publication history

The pre-publication history for this paper can be accessed here:


